# A Case Series of SARS-CoV-2 and Influenza Co-infection

**DOI:** 10.7759/cureus.17597

**Published:** 2021-08-31

**Authors:** Ruhma Ali, Aditya Patel, Kok Hoe Chan, Sindhusha Veeraballi, Jihad Slim

**Affiliations:** 1 Internal Medicine, Saint Michael's Medical Center, Houston, USA; 2 Internal Medicine, Saint Michael's Medical Center, Newark, USA; 3 Internal Medicine, University of Texas Health Science Center at Houston, Houston, USA; 4 Medical Education, Saint Michael's Medical Center, Newark, USA; 5 Infectious Diseases, Saint Michael's Medical Center, Newark, USA

**Keywords:** covid 19, influenza, sars-cov-2 (severe acute respiratory syndrome coronavirus -2), case series, influenza and covid-19 co-infection

## Abstract

The novel coronavirus 2019, a disease associated with SARS-CoV-2 infections has resulted in significant morbidity and mortality across the globe. In the United States, influenza has been one of the leading causes of hospitalization during the winter season. To date, the co-infection of SARS-CoV-2 and influenza virus has created a unique challenge for healthcare workers, especially during the cold season. Both viruses have similar clinical presentation and transmission characteristics. Many reports are available for either SARS-CoV-2 and influenza individual infections, but limited data are available for the co-infection. Herein, we present a case series of five cases of SARS-CoV-2 and influenza co-infection as well as their clinical characteristics, laboratory findings, management, and outcome.

## Introduction

The 2019 novel severe acute respiratory syndrome coronavirus (SARS-CoV-2) has affected 188,655,968 worldwide with over 4,067,517 deaths till July 2021 [1]. Current evidence suggests that the causative agent of coronavirus 2019 (COVID-19) is primarily transmitted through contact and respiratory droplets similar to the influenza virus. The clinical presentation of COVID-19 is also similar to the influenza virus. Influenza has been known to cause infection with other respiratory pathogens [2]. However, limited data is available on the morbidity and mortality of patients with COVID-19 and influenza co-infection. In this case series, we presented five patients who were admitted with COVID-19 and influenza co-infections as well as their clinical outcome and treatment plan.

## Case presentation

Patient 1 

**Figure 1:**
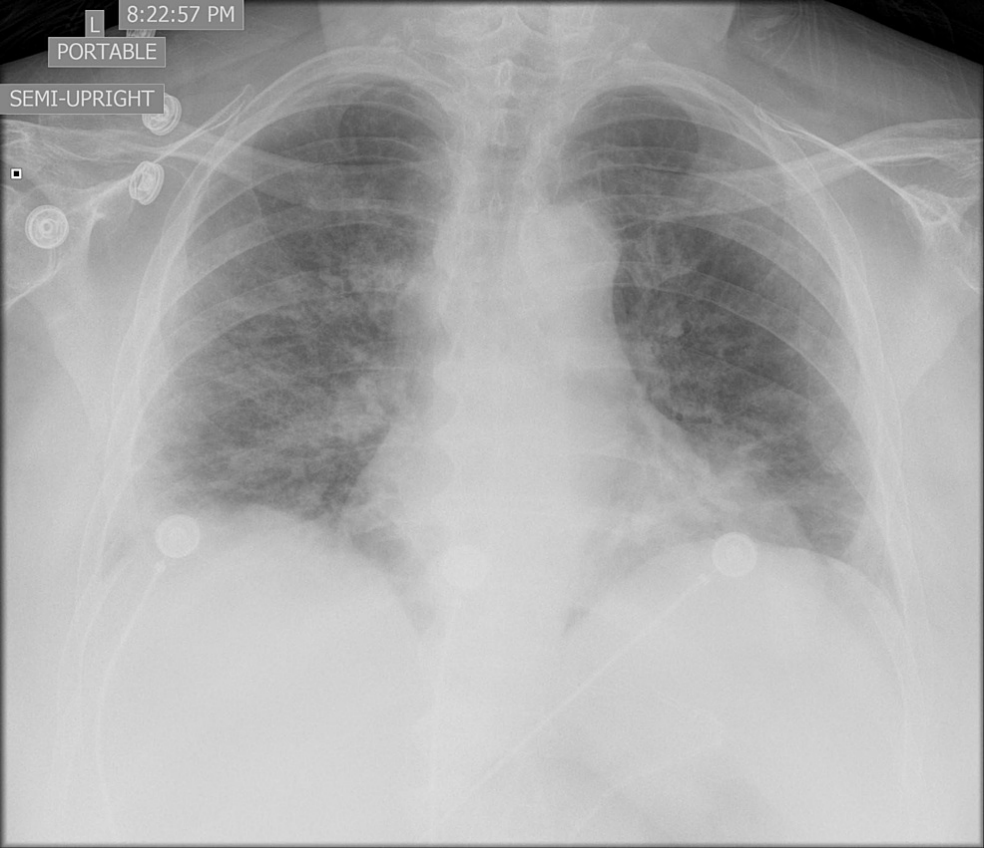
Chest x-ray which showed bilateral diffuse consolidation consistent with multifocal pneumonia

A 61-year-old female with a past medical history of asthma and a recent diagnosis of COVID-19 (five days prior) presented to the hospital with complaints of cough, fever, and worsened shortness of breath for three days. She had a positive tuberculosis skin test 10 years ago, she denied any symptoms and has not received isoniazid. She was a former smoker, quit 23 years ago. On admission, her body temperature was 97.9 ⁰F, blood pressure (BP) 128/74 mmHg, heart rate (HR) 78 beats/min, respiratory rate (RR) 19 breaths/min, and saturating 93% on room air (RA). Physical examination was notable for decreased breath sounds with crackles heard at the bases bilaterally. Chest x-ray (CXR) showed diffuse bilateral consolidation consistent with multifocal pneumonia (Figure 1). Electrocardiogram (ECG) was remarkable and troponin, as well as brain natriuretic peptide, were negative.

SARS-CoV-2 reverse transcription-polymerase chain reaction (RT-PCR), antigen, and Immunoglobulin M/Immunoglobulin (IgM/IgG) antibodies were positive. Rapid influenza A was also positive. All the inflammatory markers were elevated. The patient required oxygen supplementation in the hospital. She received remdesivir for three days, dexamethasone for four days, and therapeutic anticoagulation while she was an inpatient and was discharged on oseltamivir for five days and dexamethasone for six days along with home oxygen. 

Patient 2

**Figure 2:**
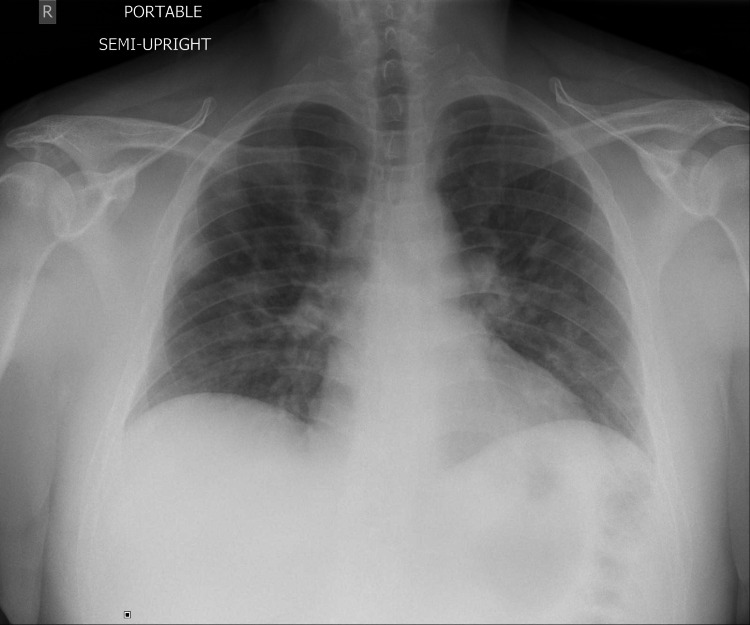
Bilateral pulmonary opacities compatible with multifocal infection or edema.

A 33-year-old male with no significant past medical history tested positive for SARS-CoV-2 RT-PCR six days before presenting to the emergency department with complaints of cough, non-bloody diarrhea, lack of appetite, and exertional shortness of breath for four days duration. On admission, the temperature was 98 ⁰F, BP 125/84, HR 104, RR 25 saturating 90% on RA. Physical examination was unremarkable. Both SARS-CoV-2 RT-PCR and antigen were positive. Rapid influenza test was also positive for influenza B. CXR showed bilateral pulmonary opacities. Inflammatory markers were mildly elevated (Figure 2). The patient received prophylactic anticoagulation, five days of remdesivir, and seven days of dexamethasone. His saturation improved and he was discharged home. 

Patient 3

**Figure 3:**
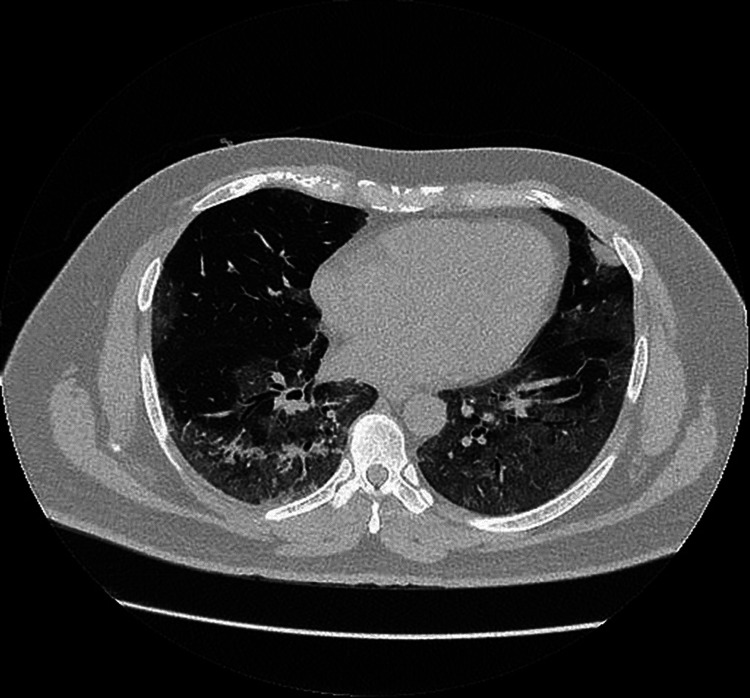
Computed tomography (CT) chest  demonstrated bilateral extensive ground-glass opacification at the peripheral lung zones

A 46-year-old male presented with fever, productive, non-bloody cough associated with a right earache, decreased appetite, double vision, and myalgias for eight days duration. He was tested positive for SARS-CoV-2 five days prior to admission. He recently finished a five-day course of azithromycin. On admission, his temperature was 101.5 ⁰F, BP 121/92, HR 109, RR 19, and saturating 94% on RA. Physical examination notable for rhonchi at the lower lung bases bilaterally on respiratory examination. CXR showed patchy left lower lung density possibly due to atelectasis. Computed tomography (CT) chest demonstrated bilateral extensive ground-glass opacification at the peripheral lung zones (Figure 3). Both SARS-CoV-2 RT-PCR and antigen were tested positive. The rapid influenza antigen test was negative, however, the influenza molecular test was positive for influenza B. The inflammatory markers were also elevated. He received five days of oseltamivir, five days of remdesivir, and 10 days of dexamethasone. He was also given 1 unit of convalescent plasma in light of the severe COVID-19 with cytokine storm and respiratory distress. He did not require mechanical ventilation. He was discharged on home oxygen and anticoagulation for two weeks.

Patient 4 

**Figure 4:**
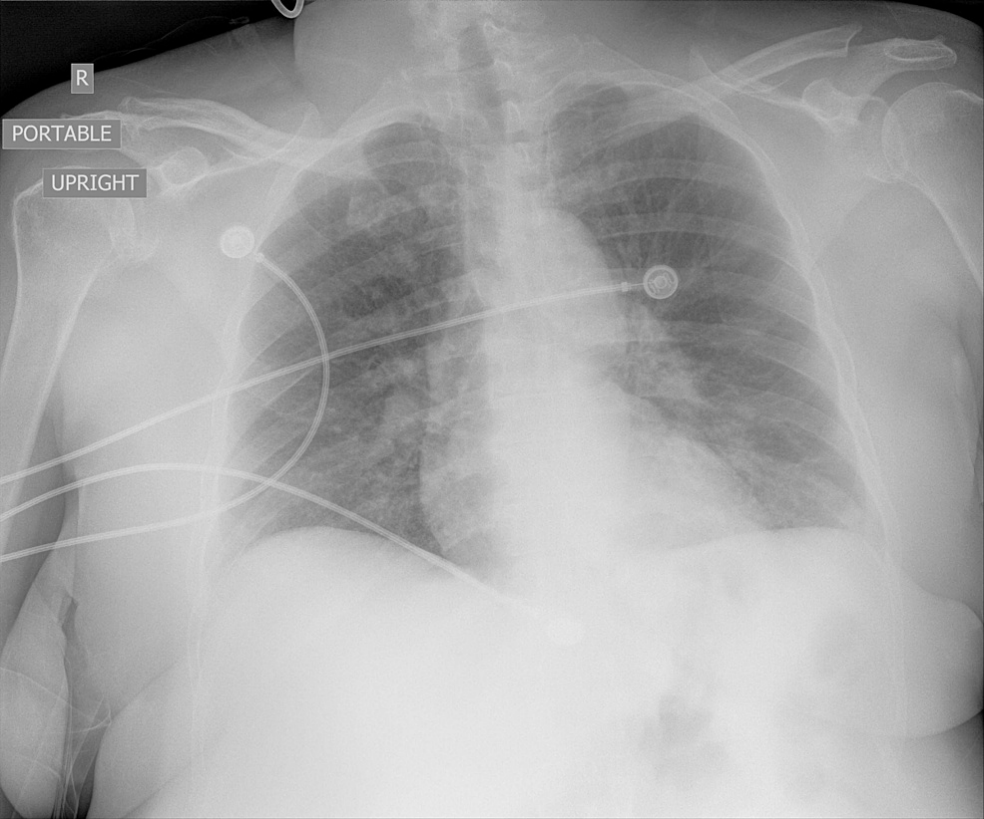
Mild diffuse bronchial wall thickening without focal consolidation or significant pleural fluid

A 57-year-old female with a past medical history significant for chronic obstructive pulmonary disease was initially admitted to the intensive care unit for dizziness and nausea secondary to hyponatremia with a serum sodium level of 116 mmol/L. She was admitted a year ago for acute hypoxic respiratory failure secondary to influenza A and B. On admission, her temperature was 98.7 ⁰F, BP 143/90, HR 90, RR 14, and saturating 92% on RA. On physical examination, bilateral rales were heard. CXR was significant for mild diffuse bronchial wall thickening (Figure 4). SARS-CoV-2 rapid antigen was positive with elevated inflammatory markers consistent with COVID-19, however, both RT-PCR and IgM/IgG antibodies were negative. Rapid influenza test was also positive for influenza B. She was given three days of remdesivir, five days of oseltamivir, and therapeutic anticoagulation. She did not require mechanical ventilation and was discharged home on two weeks of anticoagulation.

Patient 5

**Figure 5:**
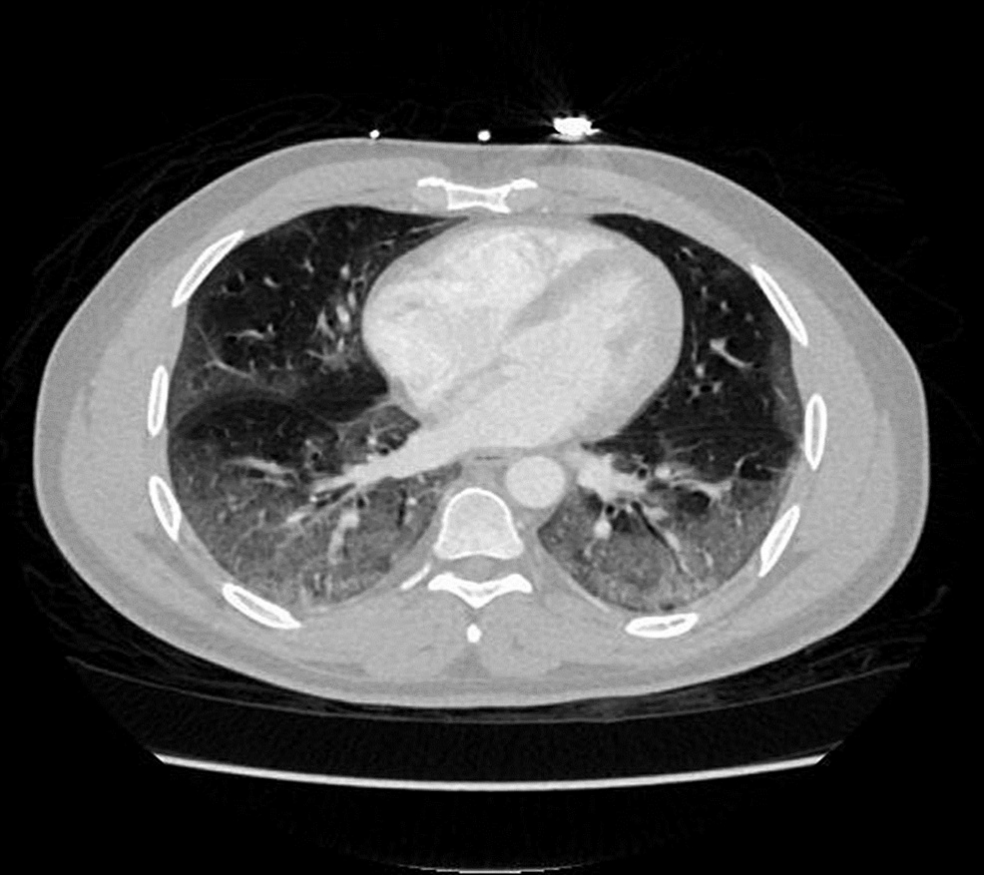
Bilateral multi-lobar peripheral dominant ground-glass opacities

A 41-year-old African American male with no significant past medical history presented with complaints of fever, loss of appetite, myalgia, non-bloody, non-bilious vomiting, and shortness of breath for three days duration. On admission, his temperature was 98.9 ⁰F, BP 122/72, HR 87, RR 25, and saturating 87% on RA. Physical examination was notable for bilateral diffuse crackles throughout the lungs. CXR showed diffuse and patchy bilateral infiltrates. CT scan showed bilateral multi-lobar peripheral dominant ground-glass opacities (Figure 5). Both SARS-CoV-2 antigen and RT-PCR were tested positive. Rapid influenza B antigen was also positive. His inflammatory markers were elevated. He was transferred to the intensive care unit for increasing oxygen requirements. He required mechanical ventilation and was also started on pressor support due to hypotension. His blood culture was positive for fusobacterium necrophorum caused by dental erosion as seen on the physical examination. He was given ceftriaxone and metronidazole for five days. He received remdesivir for five days, dexamethasone for 10 days, and oseltamivir for 10 days. He also received convalescent plasma for COVID 19. He developed multiorgan failure in the ICU with worsening renal and liver function. The patient expired due to cardiac arrest on the 20th day of admission.

## Discussion

The co-infection of SARS-CoV-2 and influenza has created a unique challenge for health care workers. Co-infection refers to simultaneous infections of the host by multiple pathogens of the same class (example viruses) or different classes (for example viruses with bacteria or fungus) [3]. Multiple examples of this complex phenomenon exist including hepatitis D co-infection with hepatitis B, the interaction of tuberculosis with human immunodeficiency virus (HIV) in acquired immunodeficiency syndrome (AIDS) as well as co-infection of respiratory syncytial virus (RSV) and human metapneumovirus (hMPV) [2-3]. COVID-19 co-circulates in the environment with other respiratory pathogens including influenza. In this case series, we are interested to look at the clinical spectrum and disease severity of SARS-CoV-2 infection with influenza virus. 

The disease burden due to laboratory-confirmed influenza, as of April 10, 2021, has been lower as compared to the previous years. In the 2019 to 2020 periods, 39 to 56 million influenza cases with 410,000 to 740,000 hospitalizations were recorded by the center of disease control, whereas between October 1, 2020, and April 30, 2021, only 224 confirmed cases of influenza were reported with a cumulative hospitalization rate of 0.8 per 100,000 population [4]. This could be attributed to the preventive measures taken for COVID-19 including social distancing, hand hygiene, and use of face masks or it could just be under-testing or under-reporting.

The clinical presentation of both pathogens can be highly similar. COVID-19 primarily affects the lungs and can lead to multiorgan failure and death, similar to influenza. In this case series, five patients co-infected with COVID-19 and influenza had complaints of cough, myalgia, loss of appetite, and shortness of breath. The laboratory findings revealed lymphocytopenia in four patients and elevated CRP in five patients, elevated d-dimer in three patients, and elevated ferritin in four patients. The renal function was affected in one patient who later required dialysis. The detailed demographic, clinical characteristic, and inflammatory markers were demonstrated in Table 1.

**Table 1:** Demographics and baseline laboratory data

	Case 1	Case 2	Case 3	Case 4	Case 5	Reference values
Age in years	62	34	46	57	41	
Gender	Female	Male	Male	Female	Male	
Body mass index (kg/m2)	34.2	34.2	33.6	31.8		
Race/ethnicity	White/Latino	White/Latino	White/Non-Hispanic	White/Non-Hispanic	African American	
Co-morbidity	Asthma	None	Diabetes mellitus, Hyperlipidemia	COPD, Hypertension, Diabetes mellitus	None	
Intensive care unit admission	No	No	No	Yes	Yes	
Blood urea nitrogen	10	7	17	18	15	7-18 mg/dl
Serum creatinine	0.6	0.7	1.1	0.7	1.4	0.5-1.2 mg/dl
Albumin	2.7	3.6	2.8	3.1	3.4	3.4-5 mg/dl
Total protein	7.6	7.8	7.8	7	7.5	6.4-8.6 g/dl
Aspartate aminotransferase	62	31	66	54	98	15-37 U/L
Alanine aminotransferase	35	33	51	59	65	16-61 U/L
Alkaline phosphatase	97	52	47	189	55	45-117 U/L
Total bilirubin levels	0.6	0.4	0.4	1	0.7	0.2-1.0 mg/dl
White blood cells	4.7	5	3.9	18.7	4.8	4.0-11K/µL
Hemoglobin	13.3	13.7	13.8	12.8	16.4	11.2-15.7 g/dl
Hematocrit	38.8	41.3	40.9	37.5	46.4	34.1-44.9 %
Platelet	251	227	130	93	98	150-400 K/µL
Absolute lymphocyte	700	700	800	2700	600	1180-3740 /µL
Creatinine kinase (CK)	66	347	269	553	-	26-192 U/L
Lactate dehydrogenase (LDH)	482	327	510	288	453	84-246 U/L
C-reactive protein (CRP)	10.7	87	11.8	35.3	4.4	0.00-1.00 mg/dl
Ferritin	345	52.6	1,240	988	959.4	8.0-252 ng/ml
D-dimer	1042	247	417	4.512	991	0.00-0.39 µg/ml

One patient developed acute respiratory distress syndrome (ARDS) and required mechanical ventilation. Four patients were treated with antiviral therapy, oseltamivir, and all patients were treated with remdesivir. One patient was admitted to the intensive care unit and expired due to the severity of the disease and four patients were discharged home. Only one patient was given anticoagulation on discharge. The detailed treatment and clinical outcomes were listed in Table 2.

**Table 2:** Treatment and clinical outcome

	Case 1	Case 2	Case 3	Case 4	Case 5
Oseltamivir	Yes	No	Yes	Yes	Yes
Antibiotic therapy	No	No	No	Yes	Yes
Remdesivir	Yes	Yes	Yes	Yes	Yes
Dexamethasone	Yes	Yes	Yes	No	Yes
Length of hospital stay- days	4	6	12	3	20
Outcome	Alive	Alive	Alive	Alive	Expired

Co-infection with COVID-19 and influenza virus have been reported in different parts of the world with single cases seen in Taiwan, Brazil, Japan, Germany, and Spain at the start of the pandemic. Four cases were observed in Iran [5], five patients among 115 in Wuhan had coinfection with COVID-19 and influenza [6], six patients among 1103 in Turkey [2], and 23 cases among 105 in Northeastern Iran [7]. The clinical characteristics of patients with both COVID-19 and influenza were similar to those with COVID-19 alone. The symptoms of COVID-19 take longer to occur and the person is contagious for a longer duration. Testing is required to differentiate between the two respiratory pathogens as per Infectious Diseases Society of America (IDSA) guidelines [6]. The main complications reported were ARDS (20% of the patients), acute kidney damage, and acute liver failure (60% of the patients) [7]. In our case, ARDS was the major complication seen in one patient.

Guidelines regarding the treatment of COVID-19 are constantly evolving. Corticosteroid therapy has been shown to be effective in moderate to severe cases of COVID-19, however, it can be harmful to patients with influenza [8]. A recent preliminary report found that dexamethasone resulted in lower 28-day mortality among patients with COVID-19 respiratory support. The effect of corticosteroids on patients with pneumonia remains controversial and is restricted in the setting of clinical trials so far [9]. Several observational studies have failed to demonstrate any beneficial effects of steroid use for patients with influenza pneumonia. Further randomized controlled trials are required to support this hypothesis. Patients with influenza are usually treated with oseltamivir. Patients with co-infection should be treated with oseltamivir and standard of care of COVID-19. 

## Conclusions

Both influenza and COVID-19 can have similar presentations. It is important to recognize the co-infection since the management and prognosis will be different. Glucocorticoids should be used with caution in patients with positive influenza virus due to the negative effects of steroids on the morbidity and mortality of these patients. Nonetheless, for patients with influenza and COVID-19, they should be treated with antiviral for influenza (oseltamivir) and COVID-19 (remdesivir) with cautions use of dexamethasone.

## References

[1] (2021). WHO coronavirus (COVID-19) dashboard. https://covid19.who.int/.

[2] Ozaras R, Cirpin R, Duran A (2020). Influenza and COVID-19 coinfection: report of six cases and review of the literature. J Med Virol.

[3] Covin S, Rutherford GW (2021). Coinfection, severe acute respiratory syndrome coronavirus 2 (SARS-CoV-2), and influenza: an evolving puzzle. Clin Infect Dis.

[4] (2020). Weekly US influenza (flu): disease burden of influenza. https://www.cdc.gov/flu/weekly/index.htm.

[5] Khodamoradi Z, Moghadami M, Lotfi M (2020). Co-infection of coronavirus disease 2019 and influenza a: a report from Iran. Arch Iran Med.

[6] Ding Q, Lu P, Fan Y, Xia Y, Liu M (2020). The clinical characteristics of pneumonia patients coinfected with 2019 novel coronavirus and influenza virus in Wuhan, China. J Med Virol.

[7] Hashemi SA, Safamanesh S, Ghasemzadeh-Moghaddam H, Ghafouri M, Azimian A (2021). High prevalence of SARS-CoV-2 and influenza A virus (H1N1) coinfection in dead patients in Northeastern Iran. J Med Virol.

[8] Brun-Buisson C, Richard JC, Mercat A, Thiébaut AC, Brochard L (2011). Early corticosteroids in severe influenza A/H1N1 pneumonia and acute respiratory distress syndrome. Am J Respir Crit Care Med.

[9] Nedel WL, Nora DG, Salluh JI, Lisboa T, Póvoa P (2016). Corticosteroids for severe influenza pneumonia: a critical appraisal. World J Crit Care Med.

